# Cathelicidin LL-37 promotes EMT, migration and metastasis of hepatocellular carcinoma cells in vitro and mouse model

**DOI:** 10.1080/19336918.2023.2168231

**Published:** 2023-01-19

**Authors:** Huidan Zhang, Xueli Yuan, Yaxin Yang, Yangke Wanyan, Liping Tao, Yuqing Chen

**Affiliations:** Jiangsu Province Key Laboratory for Molecular and Medical Biotechnology, Life Sciences College, Nanjing Normal University, Nanjing, Jiangsu, China

**Keywords:** Hepatocellular carcinoma, hCAP18/LL-37, metastasis, MAPK/ERK signaling, 1,25(OH)_2_D_3_

## Abstract

The effect of cathelicidin hCAP18/LL-37 in hepatocellular carcinoma (HCC) metastasis remains unclear. Here, we confirmed that LL-37 expression enhanced endothelial-mesenchymal transition (EMT), migration and invasion in HCC cells. And the HER2/EGFR-MAPK/ERK signal participated in the process above. More frequent lung metastases were observed in an LL-37-overexpressing hematogenous metastasis model. Interestingly, 1,25(OH)_2_D_3_ together with si-LL-37 significantly enhanced 1,25(OH)_2_D_3_-induced inhibition of migration and invasion in PLC/PRF-5 cells, and also enhanced reversion of the EMT process. Therefore, LL-37 is involved in HCC metastases, and may act as an important factor to attenuate the inhibitory activity of 1,25(OH)_2_D_3_ on HCC metastasis. Targeting hCAP18/LL-37 may offer a potential strategy to improve the anticancer activity of 1,25(OH)_2_D_3_ in HCC therapy.

## Introduction

Hepatocellular carcinoma (HCC) has the third highest mortality rate among cancer patients, resulting in 830,000 deaths annually [1]. Due to unsatisfactory progress in developing effective strategies for early diagnosis and treatments, 70% to 80% of patients have already reached an advanced stage at the time of diagnosis [2]. Metastasis is one of the main reasons for the high recurrence rate and poor prognosis of HCC. Although development of intrahepatic metastases is the most common reason why patients with advanced stage HCC die of liver failure resulting from advanced intrahepatic lesions, extrahepatic metastasis (EHM) via hematogenous spread, lymphatic dissemination or direct invasion is another important cause of death in HCC patients [3]. HCC patients with EHM usually have a poor prognosis with an expected median survival time of 6 months [4]. The lung is considered the most likely organ for HCC metastatic colonization, accounting for 47% of all EHM, followed by lymph nodes, bones and adrenal glands [3,5]. To date, systemic therapy for advanced HCC includes molecular targeted therapy, immune checkpoint inhibitors or a combination of both [6]. Based on the results of recent clinical trials, it appears that a single drug may not be sufficient for the treatment of HCC. Combination therapy now represents a major research direction for the systemic treatment of advanced HCC [7]. It thus will be necessary to further our understanding of the mechanisms underlying HCC metastases in order to develop new therapeutic strategies against advanced HCC in the future.

Human cathelicidin hCAP18/LL-37 is a well-known host defense peptide secreted by various cell types, including epithelial cells and immune cells [8]. After cleavage by protease (such as neutrophil proteinase 3) and epithelial kallikreins (such as kallikrein 5), hCAP18 releases the active LL-37 peptide, which exerts multiple biological actions involving antibacterial effects, chemotaxis, wound healing, lipopolysaccharide neutralization, angiogenesis and immunomodulation [9]. Accumulating evidence indicates that LL-37 plays a significant carcinogenic role in most cancers [9–15]. Additionally, the promotion of invasion and metastases induced by LL-37 have been reported in ovarian, malignant melanoma, breast cancer and skin squamous cell carcinoma, as determined primarily from in vitro studies [16–18]. However, its role in HCC metastasis remains unknown.

Our previous research demonstrated that hCAP18/LL-37 has a promotional effect on HCC cell proliferation and tumor growth both in vitro and in vivo [19]. Furthermore, hCAP18/LL-37 expression can be significantly induced by 1,25(OH)_2_D_3_ in HCC cells and in xenograft tumor tissue, which in turn suppresses the antitumor growth activity of 1,25(OH)_2_D_3_ in HCC xenograft tumors. In addition, 1,25(OH)_2_D_3_ inhibits the migration, invasion or metastasis of several cancers including colon cancer [20], ovarian cancer [21] and prostate cancer [22] in vitro or in vivo. Moreover, 1,25(OH)_2_D_3_ or its analog prevents lung and bone metastasis, and prolonged animal survival time was also reported in a breast cancer mouse model [23,24]. Provvisiero et al. (2019) showed that 1,25(OH)_2_D_3_ prolonged everolimus-induced transition to the mesenchymal phenotype by restoring the epithelial phenotype in everolimus-resistant HCC cells in vitro, suggesting that 1,25(OH)_2_D_3_ may be involved in the endothelial-mesenchymal transition (EMT) of HCC cells [25]. However, the role of 1,25(OH)_2_D_3_ on HCC metastasis, and the effect of hCAP18/LL-37 expression on the anti-metastatic effects of 1,25(OH)_2_D_3_ remains unknown.

EMT is a transformation process mandatory for the local and distant progression of many malignant tumors including HCC [26]. Usually, epithelial cells lose their characteristic marker E-cadherin and gain mesenchymal markers (such as N-cadherin and Vimentin) during the EMT process [27]. During the process, a number of transcription factors (such as Snail, Slug and Twist) are involved in the EMT of HCC, and their presence is associated with a poor prognosis [28]. Similar to most cancers, HCC tumor invasion and metastasis depend to a large extent on the proteolytic activity of a large number of matrix metalloproteinases (MMPs) which affect cell-to-cell and cell-matrix communication [29]. MMP levels and the activation of EMT-related transcription factors are controlled by several signaling pathways, which participate in migration, invasion and metastasis. Among these pathways, the mitogen-activated protein kinase (MAPK) pathway plays an indispensable role in the development of EMT in a variety of cancer cells and is closely related to the malignant behavior of tumors [30,31]. Our previous study showed that the MAPK pathway was significantly enriched in LL-37-overexpressing HCC cells [19]. However, whether MAPK pathway is involved in migration and invasion of HCC cells in the anticancer activity of LL-37 remained unclear.

In the present study, we analyzed the effects of LL-37 expression on EMT, migration and metastasis in cultured HCC cells and in HCC xenograft and hematogenous metastasis mouse models. We also assessed the effect of LL-37 expression on 1,25(OH)_2_D_3_-mediated regulation of EMT, migration and invasion by HCC cells. Our findings revealed the role of hCAP18/LL-37 in migration and metastases of HCC cells, and may also help to develop an effective anticancer strategy for 1,25(OH)_2_D_3_ in HCC treatment.

## Materials and methods

### Chemicals and cell culture

LL-37 peptide was synthesized by Synpeptide Inc (Nanjing, China). We purchased 1,25(OH)_2_D_3_, KO-974 and Neratinib from MedChemExpress (NJ, USA). Protein A/G and primary antibodies against hCAP18/LL-37, ERK1/2, p-ERK1/2, MMP14, MMP9, N-cadherin, E-cadherin, Vimentin, Slug, VDR, EGFR, p-EGFR (Tyr845) and Snail were obtained from Santa Cruz (CA, USA). Antibodies against β-actin and p-HER2 (Tyr1248) were purchased from Abclonal (Wuhan, China), as well as anti-rabbit and anti-mouse secondary antibodies. Anti-flag was purchased from Proteintech (Wuhan, China). HER2 antibody was purchased from Cell Signaling Technology (MA, USA). The human HCC cell lines Huh7 and PLC/PRF-5 were purchased from American Tissue Culture Collection (ATCC, Shanghai, China). Cells were maintained in DMEM (Thermo Fisher Scientific, USA) supplemented with 10% fetal bovine serum (FBS), penicillin (100 U/mL) and streptomycin (100 mg/mL) (Solarbio, Beijing, China) at 37°C in 5% CO_2_. We acquired 3,3’-diaminobenzidine (DAB) from Beyotime (Nanjing, China). All other reagents were analytical grade reagents and produced in China.

### Plasmids construction and cell transfection

The LL-37 coding sequences (GenBank accession no. 820) was cloned from the cDNA of human L02 cells using Oligo dT23 primers including a pair of *Bam HI* and *Xbal I* restriction sites. Amplify the LL-37 coding sequence, and the eukaryotic expression vector pcDNA3.0-LL-37 was constructed and then transformed into *DH5α E. coli*. The orientation of the pcDNA3.0-LL-37 was verified by sequencing. The plasmid was transfected into HCC cells using the HiTrans^TM^ LipoPlus reagent. Briefly, cells (3 × 10^5^ cells/mL) were seeded into six-well plates and incubated for 12 h until they reached 80–90% confluence. 2 μg of pcDNA3.0, pcDNA/LL-37, si-control (scrambled control RNA), and si-LL-37 (5’-GTCCAGAGAATCAAGGATT-3’) were added, respectively. After 24 h, to select for transfected cells, 800 µg/ml G418 was administered for 3–5 days and until antibiotic-resistant colonies were observed. Selection of recombinant transfectants was performed for at least 30 days. Finally, the constructed stable PLC/PRF-5^LL−37^ cells were identified by qRT-PCR and western blotting.

### Western blot analysis

Total proteins were extracted using RIPA containing PMSF and phosphatase inhibitors (Beyotime, Shanghai, China). Protein concentration was determined with a BCA Kit (Vazyme, Nanjing, China). A total of 20 μg of protein was loaded per lane, separated by 8–15% SDS-PAGE and transferred to PVDF membranes (Millipore, Darmstadt, Germany). Subsequently, the membranes were blocked and incubated with primary antibodies (1:1000) overnight at 4°C and then were incubated with HRP-conjugated secondary antibodies for 1 h at room temperature. The visualization of bands was detected using an ECL detection system (Tanon, GE, USA) and analyzed with Image J densitometry analysis software (NIH).

### Wound healing assays

Wound healing assays were used to assess cell migration. PLC/PRF-5 and Huh7 cells (~2 × 10^5^) were plated in 12-well plates. The monolayers were scratched with a 200 μl sterile pipette tip. The cells were washed with PBS to remove non-adherent cells. Subsequently, the cells were cultured in serum-free DMEM. The wound surface was observed under light microscope at 0 h, 48 h and 72 h. The width of the scratched gaps at 0, 48 h and 72 h was measured using Image J software. The wound closure rate was calculated using the following formula: Wound closure rate (%) = (Original width−Width after migration)/Original width×100. Each independent experiment was repeated four times.

### Transwell assays

Transwell analysis was used to determine cell invasive capacity. Briefly, transwell chambers with 8-μm pore size (Corning, USA) were coated with 100 μl of 1:8 diluted Matrigel (BD Biosciences, USA) and incubated at 37°C for 4 h. The transfected cells (0.5–1 × 10^5^ cells) were cultured in the upper chamber with DMEM supplemented with 1% FBS. Then, 500 μl of DMEM supplemented with 15% FBS were added to the lower chamber. After incubation for 24 h, the cells in the upper chamber were removed with a cotton swab. The cells in the lower chamber were fixed using 4% paraformaldehyde and stained with 0.1% crystal violet. After imaging the cells under a light microscope, the cells were eluted with 33% acetic acid and detected at 570 nm with a microplate reader to calculate the invasion rate. Each independent experiment was replicated at least four times.

### Real-time quantitative polymerase chain reaction (qRT-PCR)

Total RNA was extracted from cells using TRIzol reagent (Vazyme, China) according to the manufacturer’s protocols. RNA was quantified using a NanoDrop ND-1000 spectrometer. Then, RNA was reverse-transcribed into cDNA using HiScriptIII RT SuperMix for qPCR (+gDNA wiper) (Vazyme, China). The relative expression of genes was examined using AceQ qPCR SYBR Green Master Mix (Vazyme, China). Gene expression was calculated using the 2^−ΔΔCt^ method. The primers used in this study are listed in Table 1.

**Table 1.** The primers for qRT-PCR.

Primer	Sequence (5’-3’)
E-cadherin	F: CTTGCGGAAGTCAGTTCAGA
	R: CACCGTGAACGTGTAGCTCT
N-cadherin	F: TCAGGCGTCTGTAGAGGCTT
	R: CCAGTCTTGCATAATGCGATTTC
Vimentin	F: GCAGGAGGCAGAAGAATGGT
	R: CCACTTCACAGGTGAGGGAC
Slug	F: AATCGGAAGCCTAACTACAGCG
	R: GTCCCAGATGAGCATTGGCA
Snail	F: GTATCTCTATGAGAGTTACTCCATGCCTG
	R: TTACATCAGAATGGGTCTGCAGATGAGC
GAPDH	F: GAAGGTGAAGGTCGGAGTC
	R: GAAGATGGTGATGGGATTTC

### Immunofluorescence (IF) staining

PLC/PRF-5 cells were cultured on cell slides for 24 h, followed by treatment with 1,25(OH)_2_D_3_ (200 nM) for 24 h , then fixed with 4% paraformaldehyde for 15 min and permeabilized with 0.2% Triton X-100 for 15 min. Cells were incubated with goat anti-rabbit and anti-mouse conjugated antibodies at room temperature for 1 h in the dark, followed by counterstaining with DAPI for 30 min at room temperature. Fluorescence images were collected with a Ti-E-A1R confocal laser microscope (Nikon, Japan).

### Hematogenous metastasis model by intravenous HCC cell injection

BALB/c nude mice (4–6) weeks old were housed in specific pathogen-free conditions, and were randomly divided into three groups (6 mice per group). Then PLC/PRF-5 cells (5 × 10^6^ cells) or PLC/PRF-5^LL−37^ cells (5 × 10^6^ cells) were injected into mice of each group via the tail vein. The remaining six mice were injected with PBS as a control. Mouse body weights were measured every week. After 13 weeks, the mice were euthanatized and dissected. The lungs and livers were harvested at necropsy and fixed in 4% paraformaldehyde. The fixed lung and liver tissues were paraffin-embedded for hematoxylin/eosin (HE) staining and analyzed for the presence of metastasis.

### Xenograft mouse model

The xenograft animal assays were carried out in two batches to assess (1) the effect of LL-37 expression on EMT, and (2) the effect of LL-37 on EMT regulated by 1,25(OH)_2_D_3_. The establishment of the HCC mouse xenograft model and the different treatments were described in our previous paper [19]. After 28 days, the mice were euthanatized and the tumors were removed for western blot analysis or for preparation of paraffin sections to detect changes in EMT markers.

### HE staining

Tissues were fixed in 4% paraformaldehyde followed by paraffin embedding, and then cut into 7-μm-thick sections. Longitudinal slices were dewaxed and rehydrated, then stained with hematoxylin solution for 5 min. The sections were then stained for 5 min with 1% acid ethanol, rinsed with distilled water, and stained with eosin solution for 3 min. Finally, the sections were dehydrated with graded alcohol and cleared with xylene. Representative images were obtained using an Olympus IX51 fluorescence microscope (Nikon, Japan).

### Immunohistochemical (IHC) staining

IHC was performed using an SABC-AP kit (BOSTER, USA). The sections were dewaxed and rehydrated, then pretreated with sodium citrate for antigen retrieval. The sections were rinsed with PBS three times and then 5% bovine serum albumin was used to block nonspecific staining at 37°C for 30 min, followed by the appropriate primary antibody incubated overnight at 4°C, secondary antibody incubated at room temperature for 30 min, and incubation with SABC at room temperature for 30 min. After three washes with PBS, DAB was used for color reaction and hematoxylin solution was used for nuclear counterstaining. The sections were dehydrated in gradient ethanol, made transparent with xylene and neutral adhesive sealing compound was applied. Representative images were obtained using an Olympus IX51 fluorescence microscope.

## Statistical analysis

Values were expressed as means ± SEM from four to six independent experiments. Two-tailed Student’s t-test and one-way ANOVA with Tukey’s multiple comparison test were used to determine the significance of differences. For all cases, p < 0.05 was considered statistically significant. Statistical analysis was assessed using Statistical Package for the Social Sciences (SPSS/PC 20.0, Chicago, USA).

## Results

### LL-37 expression promoted migration and invasion of cultured HCC cells

**Figure 1.**
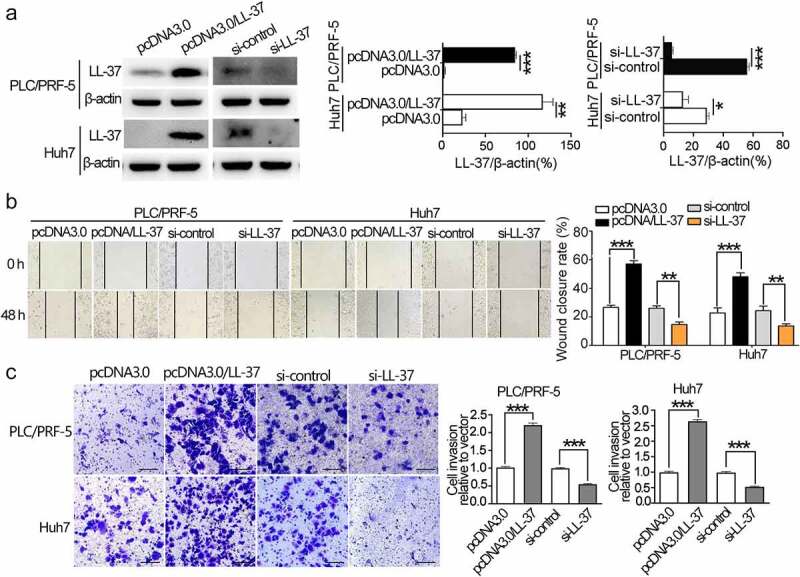
LL-37 expression promoted the migration and invasion of HCC cells in vitro. PLC/PRF-5 and Huh7 cells were transfected with pcDNA3.0, pcDNA/LL-37, si-control and si-LL-37, respectively. (a) Confirmation of LL-37 overexpression (pcDNA3.0/LL-37) and knockdown (si-LL-37) in PLC/PRF-5 or Huh7 cell by western blot. (b) The effect of LL-37 expression on migration was detected by wound healing assay. Represent images were taken at 0 h and 48 h, and the wound closure (%) was calculated from 4 independent experiments, and expressed as mean ± SEM. (c) The effect of LL-37 expression on invasion of PLC/PRF-5 or Huh7 cells was detected by transwell assays. Represent images at 24 h post-incubation and relative invasion cells were shown. Data are represented as the mean ± SEM of 4 independent experiments. ns, no significant. *p < 0.05, **p < 0.01, ***p < 0.001.

To determine whether the expression of LL-37 affects HCC cell migration and invasion, overexpression and knockdown systems were first established. The LL-37 level was significantly increased or decreased after transfection with pcDNA3.0/LL-37 or si-LL-37 for 48 h in HCC cells, respectively (Figure 1a). The wound healing assay showed that LL-37 overexpression significantly promoted migration of both PLC/PRF-5 and Huh7 cells (p < 0.01), whereas LL-37 knockdown significantly inhibited migration (p < 0.05, Figure 1b). Consistently, LL-37 overexpression also significantly promoted invasion of PLC/PRF-5 and Huh7 cells (p < 0.001), while the invasive ability of HCC cells after LL-37 knockdown was significantly impaired (p < 0.001, Figure 1c). Taken together, these data demonstrated that LL-37 could significantly promote migration and invasion of cultured HCC cells.

### LL-37 promoted EMT of HCC cells in vitro

**Figure 2.**
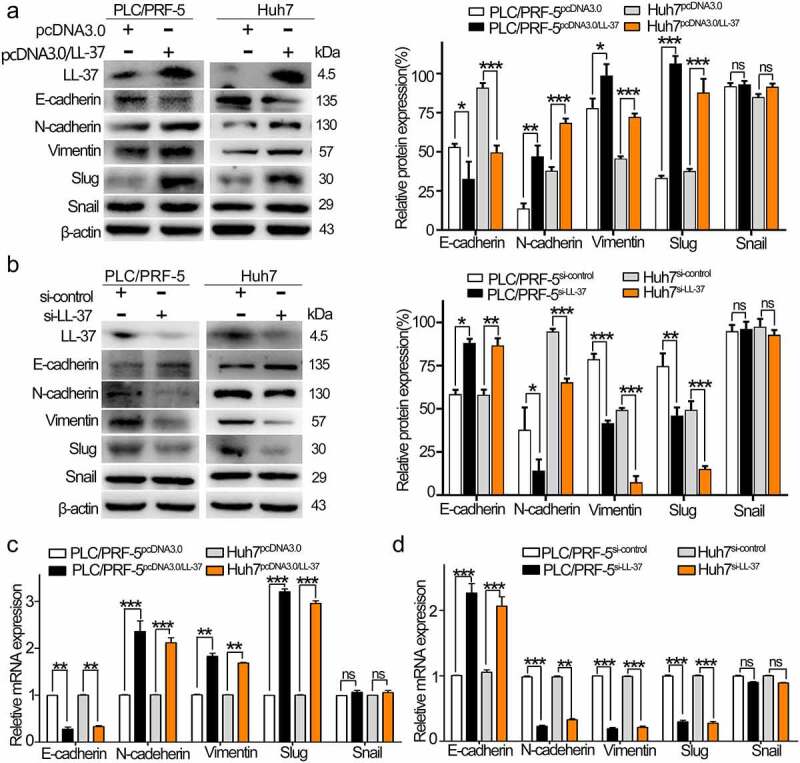
LL-37 promoted the EMT of HCC in vitro. PLC/PRF-5 and Huh7 cells were transfected with pcDNA3.0, pcDNA/LL-37, si-control and si-LL-37, respectively. (a,b) The protein levels of Vimentin, E-cadherin, N-cadherin, Slug and Snail were detected in the LL-37-overexpression HCC cells (a) or LL-37-knockdown HCC cells (b) by western blotting. (c,d) The mRNA levels of Vimentin, E-cadherin, N-cadherin, Slug and Snail in the LL-37-overexpression HCC cells (c) or LL-37-knockdown HCC cells (d) were detected by qRT-PCR assay. Data are represented as the mean ± SEM of 4 independent experiments. ns, no significance. *p < 0.05, **p < 0.01, ***p < 0.001.

In order to determine the effect of LL-37 on EMT of HCC cells, the levels of mesenchymal markers (N-cadherin and Vimentin), epithelial marker (E-cadherin) and EMT-related transcription factors (Snail and Slug) were detected. After overexpression of LL-37 in PLC/PRF-5 or Huh7 cells for 48 h, western blot assays showed that the levels of N-cadherin, Vimentin and Slug were obviously increased, while E-cadherin levels decreased significantly (Figure 2a). After knockdown of LL-37 by si-LL-37, the levels of N-cadherin, Vimentin and Slug were significantly down-regulated, while E-cadherin was significantly up-regulated (Figure 2b). qRT-PCR analysis further found that the mRNA levels of N-cadherin, Vimentin and Slug were significantly up-regulated in LL-37-overexpressing HCC cells (Figure 2c). On the contrary, the mRNA level of E-cadherin was significantly down-regulated by si-LL-37 treatment of HCC cells (Figure 2d). However, no significant change was observed in the Snail level either by overexpression or knockdown of LL-37. These results indicated that LL-37 promoted EMT in PLC/PRF-5 and Huh7 cells.

### HER2/EGFR-MAPK/ERK signaling participated in LL-37-induced EMT, migration and invasion

**Figure 3.**
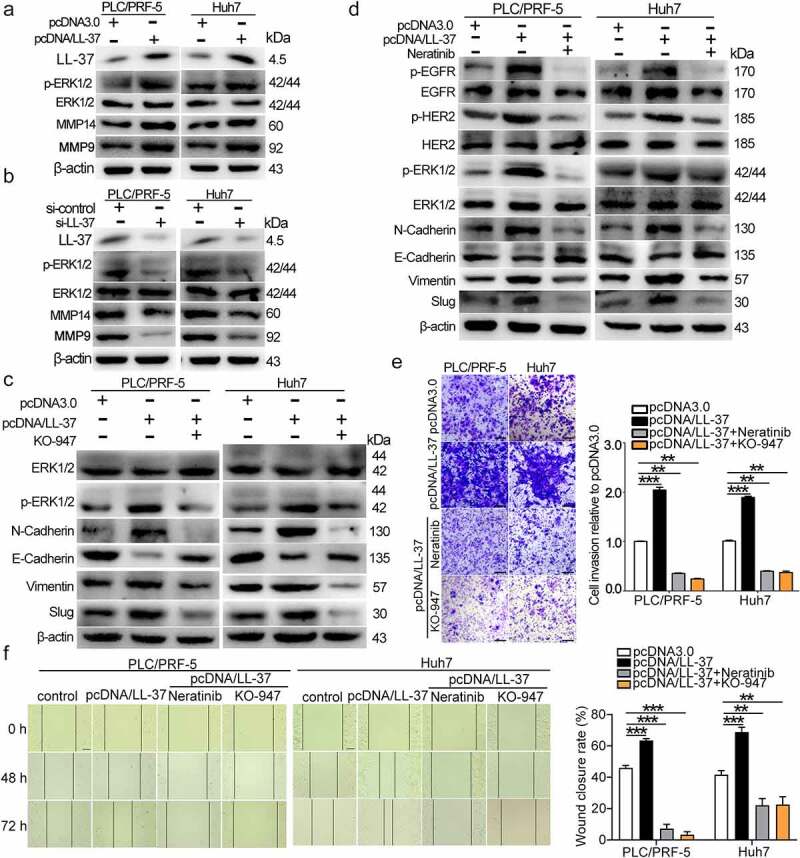
LL-37 promoted EMT via HER2/EGFR-MAPK/ERK signaling in HCC cells. PLC/PRF-5 and Huh7 cells were transfected with pcDNA/LL-37 (a), or si-LL-37 (b), and the MMP14, MMP9 and p-ERK1/2 level was detected by western blot. PLC/PRF-5 and Huh7 cells were transfected with pcDNA/LL-37 for 48 h, then KO-947 (c) or Neratinib (d) was added. Western blots detected the levels of p-ERK1/2, ERK1/2, Vimentin, E-cadherin, N-cadherin, Slug and Snail. Transwell assay (e) and wound healing assay (f) were conducted to evaluate changes in invasion and migration after KO-947 or neratinib treatment, respectively. Multiple of invasion cells and the wound closure (%) compared with control (pcDNA3.0) were calculated from 4 independent experiments. Data are represented as the mean ± SEM. **p < 0.01, ***p < 0.001.

To explore the role of the MAPK pathway on EMT, migration and invasion induced by LL-37 in HCC cells, two inhibitors were used in the following study. Results showed that LL-37 overexpression significantly increased the MMP14, MMP9 and p-ERK1/2 level in both PLC/PRF-5 and Huh7 cells (Figure 3a), whereas LL-37 knockdown by si-LL-37 resulted in a decrease of the MMP14, MMP9 and p-ERK1/2 level (Figure 3b). Furthermore, KO-947, an inhibitor of ERK1/2 phosphorylation, reversed the LL-37-induced elevation of N-cadherin, Vimentin and Slug, and also reversed the LL-37-induced decrease of E-cadherin (Figure 3c). Additionally, neratinib, a dual inhibitor of EGFR/HER2, showed similar inhibitory activity as KO-947 against LL-37-induced EMT (Figure 3d). Meanwhile, cell migration and invasion of PLC/PRF-5 and Huh7 cells induced by LL-37 overexpression were reduced after KO-947 or neratinib treatment (Figure 3e, f). Therefore, the ERK pathway played an important role in LL-37-induced EMT, migration and invasion in PLC/PRF-5 and Huh7 cells. Meanwhile, EGFR/HER2, as the upstream target for LL-37, is a key factor mediating LL37-induced EMT, migration and invasion. All these data show the important role of the HER2/EGFR-MAPK/ERK signaling pathway in mediating LL-37-induced EMT, migration and invasion in HCC cells.

### Upregulation of LL-37 promoted EMT in mouse model

**Figure 4.**
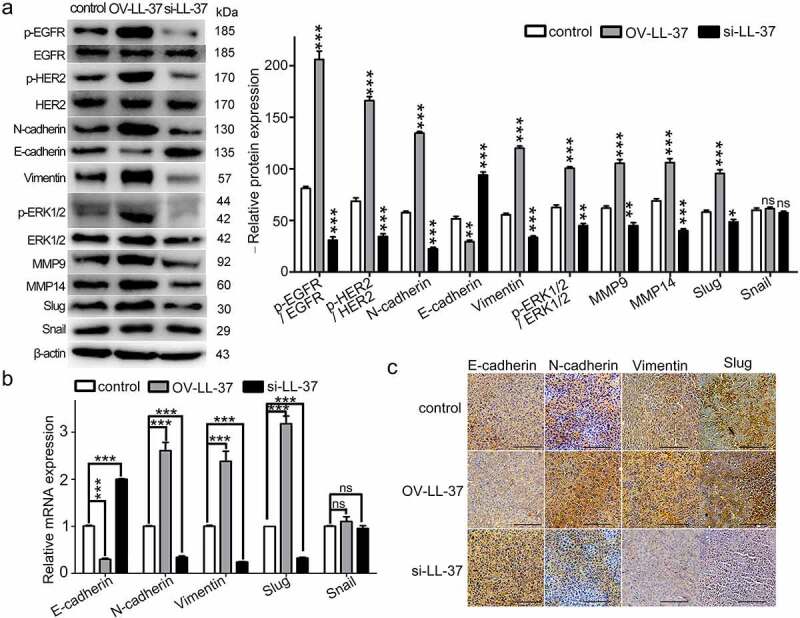
The effect of LL-37 expression on EMT in xenograft tumors. PLC/PRF-5 and PLC/PRF-5^LL−37^ (stable overexpressing LL-37 cells) were subcutaneously injected into nude mice to establish an xenograft mouse model, and the mice were assigned to three groups: PLC/PRF-5 xenografted mouse group (control group), PLC/PRF-5^LL−37^ xenograft mouse group (OV-LL-37 group) and PLC/PRF-5 xenografted mouse group with si-LL-37 treatment group (si-LL-37 group). Mice were euthanatized on day 28. (a) Tumor tissues were crushed, lysed with RIPA lysis buffer and supernatants were collected to detect p-HER2, p-EGFR, p-ERK1/2 and EMT markers by western blot. The quantified data was normalized to the protein expression of control group cells (n = 4). (b) Total RNA from tumor tissues was extracted and the expression of EMT markers was detected by qRT-PCR. (c) Tumor tissues were embedded in paraffin and sectioned for IHC staining using antibodies against EMT markers. Representative images from each group are shown. Scale bars, 50 μm. Data are represented as means ± SEM. ns, no significance. *p < 0.05, **p < 0.01, ***p < 0.001.

In order to assess the effect of LL-37 expression on EMT in vivo, we established PLC/PRF-5 xenografted mice (control), PLC/PRF-5^LL−37^ xenografted mice with stable over-expression of LL-37 (OV-LL-37), and PLC/PRF-5 xenografted mice receiving si-LL-37 treatment (si-LL-37). The expression levels of mesenchymal and epithelial markers, EMT-related transcription factors, MMPs, p-HER2, p-EGFR and p-ERK1/2 were then detected in xenograft tumor tissues. Results showed that the protein levels of N-cadherin, Vimentin, Slug, p-HER2, p-EGFR, p-ERK1/2, MMP9 and MMP14 increased significantly in PLC/PRF-5^LL−37^ xenograft tumor; while significantly decreased in PLC/PRF-5^LL−37^ xenograft receiving si-LL-37 treatment (Figure 4a). The level of E-cadherin showed the opposite change above (Figure 4a). At the mRNA level, the changes in N-cadherin, Vimentin, E-cadherin, Slug and Snail were consistent with the protein changes (Figure 4b). IHC staining further verified the increased levels of N-cadherin, Vimentin and Slug and the decreased level of E-cadherin in LL-37-overexpressing tumors (Figure 4c). Collectively, these results indicate that LL-37 is capable of promoting the EMT of HCC cells in mouse xenograft tumors.

### LL-37 overexpression promoted lung metastasis through a hematogenous pathway

**Figure 5.**
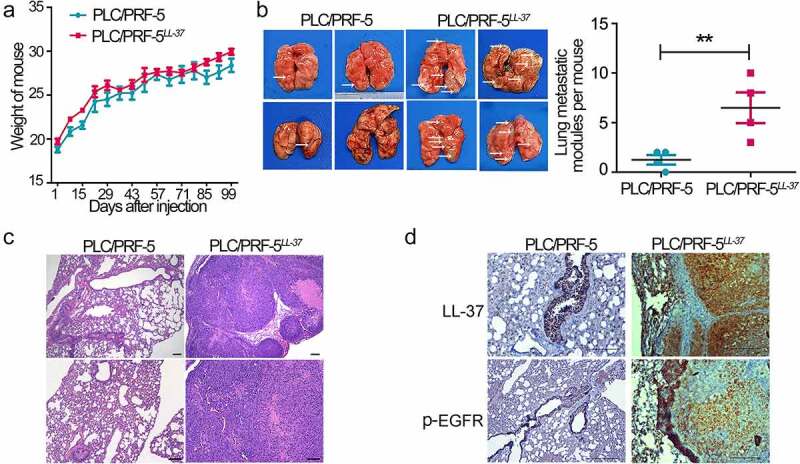
The effect of LL-37 overexpression on lung metastatic ability of HCC cells through the hematogenous approach. PLC/PRF-5 and PLC/PRF-5^LL−37^ cells were injected into nude mice (4–6 weeks old) via the tail vein. (a) Mouse weights were determined every 2 days. The mice were euthanatized after 13 weeks. (b) Representative photographs and statistical analysis of microscopically visible lung surface metastatic nodules (arrows). (c) Images of mouse lungs showing the presence of microscopic lesions visualized by HE staining. (d) Demonstration of LL-37 and EGFR in lung metastatic nodules by IHC staining using anti-hCAP18/LL-37 and anti-EGFR. Scale bars, 50 μm. Data are represented as means ± SEM. **p < 0.01.

Hematogenous metastasis is one of the main modes of HCC metastasis. To further investigate the effect of LL-37 on the hematogenous metastasis of HCC cells, a model of hematogenous metastasis in nude mice was established. The experimental mice were divided into two groups, which then received PLC/PRF-5^LL−37^ or PLC/PRF-5 cells injected via the tail vein. After 13 weeks, though no significant difference in body weight between the two groups was observed (Figure 5a), anatomic studies revealed microscopically visible metastatic nodules on the lung surface. The number of lung metastatic nodules in PLC/PRF-5^LL−37^ mice was significantly higher than in PLC/PRF-5 control mice (p < 0.01, Figure 5b). HE staining showed that LL-37-overexpressing cells (PLC/PRF-5^LL−37^) had stronger lung metastatic ability than control PLC/PRF-5 cells (Figure 5c). High levels of LL-37 and p-EGFR were confirmed in lung metastatic nodules (Figure 5d). These results indicated that LL-37 over-expression enhanced the metastatic ability of HCC cells through the hematogenous approach.

### Silencing LL-37 enhanced the inhibition of EMT, migration and invasion induced by 1,25(OH)_2_D_3_ in vitro

**Figure 6.**
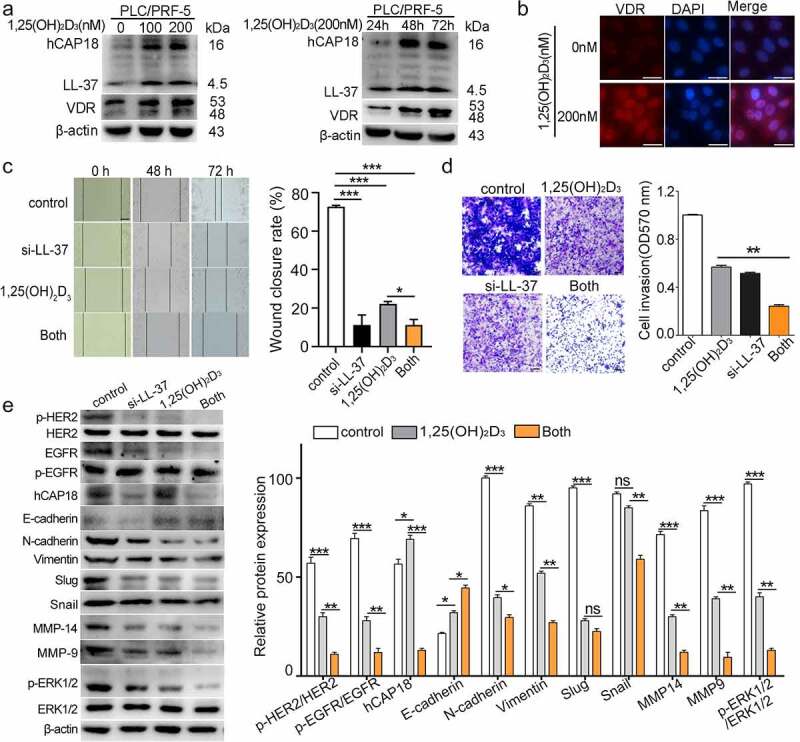
LL-37 silencing enhanced 1,25(OH)_2_D_3_-induced EMT in vitro. (a) Western blots showing detection of hCAP18, LL-37 and VDR levels in PLC/PRF-5 cells after treatment with different concentrations of 1,25(OH)_2_D_3_ (100 or 200 nM) or with 200 nM 1,25(OH)_2_D_3_ for different durations (24, 48 and 72 h). (b) After treatment with 1,25(OH)_2_D_3_ (200 nM) for 24 h, VDR distribution was detected in PLC/PRF-5 cells by immunofluorescence staining. Scale bar: 50 μm. Four treatments were performed on PLC/PRF-5 cells: control, si-LL-37, 1,25(OH)_2_D_3_ and si-LL-37 combined with 200 nM 1,25(OH)_2_D_3_. Wound healing assay (c) and Transwell assay (d) were performed with PLC/PRF-5 cells. Represent images of wound healing assay were taken at 0 h, 48 h and 72 h and transwell assay were taken at 48 h, and the wound closure (%) was calculated from 4 independent experiments, and expressed as mean ± SEM. (e) Representative EMT markers and p-ERK1/2 were assayed in PLC/PRF-5 cells by western blot. Represent images and data are represented as means ± SEM of 4 independent experiments. ns, no significance. *p < 0.05, **p < 0.01, ***p < 0.001

Our previous studies indicated that 1,25(OH)_2_D_3_ significantly induced the expression of hCAP18/LL-37 in both HCC cells and xenograft tumor tissue [19]. 1,25(OH)_2_D_3_ regulates the transcription of target genes by binding with the vitamin D receptor (VDR). Here, the obviously enhanced concentration- and time-dependent expression of hCAP18/LL-37 and VDR induced by 1,25(OH)_2_D_3_ was first confirmed in PLC/PRF-5 cells (Figure 6a). Treatment with 1,25(OH)_2_D_3_ not only up-regulated the VDR expression level, but also induced translocation of VDR into the nucleus of PLC/PRF-5 cells (Figure 6b). Meanwhile, 1,25(OH)_2_D_3_ treatment significantly inhibited the migration and invasion of PLC/PRF-5 cells (p < 0.01; Figure 6c, d). Treatment with 1,25(OH)_2_D_3_ combined with knockdown of LL-37 by si-LL-37 further enhanced its inhibitory effect on the migration and invasion of PLC/PRF-5 cells, compared with 1,25(OH)_2_D_3_ alone. In addition, 1,25(OH)_2_D_3_ treatment also inhibited EMT in PLC/PRF-5 cells, resulting in decreased levels of N-cadherin, Vimentin, Slug, MMP9 and MMP14, and increased E-cadherin level (Figure 6e). When combining 1,25(OH)_2_D_3_ with si-LL-37, the levels of N-cadherin, Vimentin, Slug, MMP9 and MMP14 were further significantly down-regulated, while the E-cadherin level was further significantly up-regulated in PLC/PRF-5 cells, compared with 1,25(OH)_2_D_3_ alone. Moreover, a very low p-HER2, p-EGFR and p-ERK1/2 levels were observed after co-treatment with 1,25(OH)_2_D_3_ and si-LL-37. Therefore, LL-37 expression induced HER2/EGFR activation, ERK1/2 phosphorylation, MMP9 and MMP14 expression, which may be involved in the impairment of 1,25(OH)_2_D_3_-induced EMT inhibition in vitro.

### Silencing LL-37 enhanced the inhibition of 1,25(OH)_2_D_3_-induced EMT in xenograft tumors

**Figure 7.**
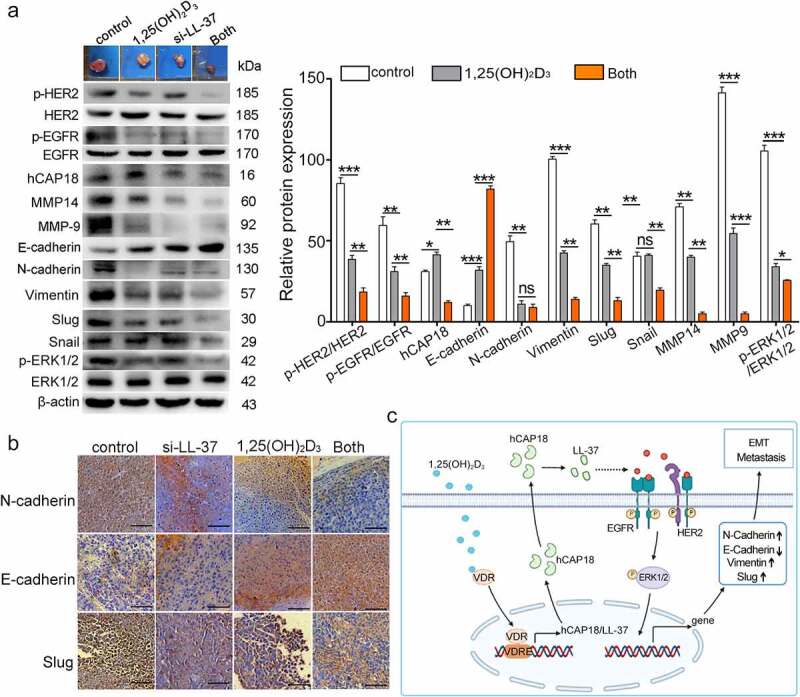
LL-37 silencing enhanced 1,25(OH)_2_D_3_-induced EMT in vivo. (a) PLC/PRF-5 xenograft tumors were crushed and lysed with RIPA lysis buffer, and then western blot detection was performed using the indicated antibodies. (b) Representative images of IHC of E-cadherin, N-cadherin and Slug in xenograft tumors. Scale bar: 50 μm. Data are represented as means ± SEM. (c) A schematic diagram summarizing the regulation mechanism of LL-37 on the EMT, migration and metastasis of HCC cells. 1,25(OH)_2_D_3_ induces the secretory expression of LL-37 through VDR receptor signal. Then LL-37 phosphorylates EGFR/HER2 to stimulate MAPK/ERK signaling, regulate the expression of EMT-related transcription factors or markers. Ultimately affects the EMT, migration and metastasis of HCC cells. ns, no significance. *p < 0.05, **p < 0.01, ***p < 0.001.

In PLC/PRF-5 xenograft tumors, 1,25(OH)_2_D_3_ treatment significantly decreased the levels of N-cadherin, Vimentin, Slug, MMP9, MMP14, p-EGFR, p-HER2 and p-ERK1/2 (Figure 7a). When combined with si-LL-37, the levels of Vimentin, Slug, MMP9, MMP14, p-EGFR, p-HER2 and p-ERK1/2 were further significantly down-regulated, while the E-cadherin level was significantly up-regulated, compared with 1,25(OH)_2_D_3_ alone. IHC staining verified the lower levels of N-cadherin and Slug and the higher level of E-cadherin in HCC tumors treated with both 1,25(OH)_2_D_3_ and si-LL-37, compared with 1,25(OH)_2_D_3_ alone (Figure 7b). These results showed that silencing LL-37 enhanced the inhibitory effect of 1,25(OH)_2_D_3_ on the EMT process both in vivo, suggesting that 1,25(OH)_2_D_3_-induced LL-37 production hampered the inhibitory effect of 1,25(OH)_2_D_3_ on EMT in vivo.

## Discussion

Accumulating studies have revealed that hCAP18/LL-37 plays a role in the promotion of tumor growth in several types of tumors through direct stimulation of malignant cells, initiation of angiogenesis or suppressing immunity in the tumor microenvironment [8,9]. However, only a few studies have reported its promotion of migration and metastasis in breast cancer [12,32], ovarian cancer cells [33] and melanoma [34]. Previously, we showed that hCAP18/LL-37 promotes the growth of HCC through the ERK/EGFR-PI3K/Akt signaling pathways [19]. Our current study further reveals the promotional effect of hCAP18/LL-37 on HCC metastasis via the ERK/EGFR-MAPK/ERK pathway both in vitro and in vivo.

Currently, because only very limited and poorly effective therapeutic options exist for HCC, this is still one of the tumors with the highest metastatic capacity and greatest risk for recurrence. EMT plays an important role in HCC metastasis and several biomarkers have been identified during this process. Here, both in cultured HCC cells and HCC xenograft tumors, LL-37 expression significantly decreased the E-cadherin level, a key biomarker for epithelial cells, and obviously increased the levels of N-cadherin and Vimentin, both biomarkers for mesenchymal cells. By losing E-cadherin-mediated cell adhesion and acquiring mesenchymal properties, carcinomatous cells acquire mobility and invasiveness, and are thus able to penetrate the surrounding stroma [35]. Although Snail is important EMT-related transcription factors in HCC, its expression was not affected by LL-37. However, the Slug level was significantly increased by LL-37 expression in HCC cells and xenograft tumors. Slug participates in EMT during cancer metastasis by binding to the promoter of downstream target genes like E-cadherin, thereby promoting the function of EMT [36]. Thus, the decreased E-cadherin level may partly result from reduced Slug induced by LL-37 in HCC cells. This implies that Slug plays an important role in LL-37-induced EMT during HCC progression.

MMPs are key factors conferring invasive and metastatic traits on malignant tumor cells by enabling their infiltration and migration in the process of EMT [29]. EMT and migration depend on increased release and activation of MMPs, as well as their cell membrane expression. Although several MMPs have been reported in HCC, secreted MMP9 is considered to be one of the most important MMPs and its functions have been well-characterized in HCC [37]. We found that LL-37 overexpression significantly increased the MMP9 level, while knockdown of LL-37 significantly decreased MMP9 levels in both HCC cells and xenograft tumors, suggesting an obvious promotional effect of LL-37 on MMP9 expression. More interestingly, membrane-type MMP14 was also significantly induced by LL-37 expression. MMP-14 has a central role among the MMPs and acts in cancer metastasis by degrading the ECM, increasing the secretion of pro-MMP2, pro-MMP9 and pro-MMP13, while cleaving membrane-anchored growth factors and cytokines [38,39]. A report showed that the increased expression of MMP14 was correlated with high rates of portal vein invasion, intrahepatic metastasis and recurrence in HCC [40].

For almost two decades, it has been known that hCAP18/LL-37 can activate EGFR signaling in a variety of cells, but the mechanisms involved are poorly understood [41]. Our previous study showed that hCAP18/LL-37 can increase HB-EGF release from membrane-anchored pro-HB-EGF and activate EGFR/HER2 in HCC cells [19]. Research further revealed that heterotrimeric G proteins regulated MMP14 directly, resulting in HB-EGF release and EGFR transactivation [42]. MMP14 activity mediated proteolytic processing to activate HB-EGF, stimulating the EGFR signaling pathway to increase proliferation and promote tumor growth [43]. One proposed model is that hCAP18/LL-37-induced G protein coupled receptors activation stimulates MMPs (such as MMP14), which subsequently cleave HB-EGF. Interestingly, Co-IP assay and MS confirmed the interaction between hCAP18 and MMP14 in PLC/PRF-5 cells (Figure S1). We speculate that hCAP18/LL-37 not only increases the expression level of MMP14, but may also be involved in the regulation of MMP14 activity to promote cell migration and invasion. Further study is needed to clarify the interactions among hCAP18/LL-37 and MMP14, which may reveal the effect of hCAP18/LL-37 on the activity of MMP14 in HCC.

Metastasis is the most lethal aspect of cancer, due to the challenges in treating the metastasis and spread of cancer to key organs. Among them, hematogenous metastasis is usually the main cause of death related to HCC, and the most common sites of hematogenous metastases are lung (in up to 60% of patients who have metastatic disease) and bone (in up to 40% of patients) [44,45]. The intravenous injection (tail vein injection) method is frequently used to generate lung metastasis models [46]. Metastatic colonization of distant tissues is a key process in tumor metastasis [47]. In this study, using a hematogenous metastasis model, we found more lung metastatic nodules in PLC/PRF-5^LL−37^-injected mice than in PLC/PRF-5 control mice, suggesting stronger lung colonization ability induced by LL-37 overexpression. Subcutaneous tumor models are also widely used in pre-clinical cancer metastasis research. An early study observed a significant increase in metastases in a xenograft tumor model using hCAP18-overexpressing breast cancer MJ1105 cells [12]. In our HCC xenograft tumor model, we observed that LL-37 expression significantly promoted the EMT process. More importantly, knockdown of LL-37 significant inhibited EMT in xenografted mice. Therefore, cathelicidin LL-37 is involved in HCC metastases by EMT.

Several pathways have been implicated in the progression of EMT in HCC, such as the Wnt/β-catenin, c-Met/HGF/Snail, Notch-1/NF-κB, TGF-β/SMAD and basic fibroblast growth factor-related signaling pathways [48]. Here we found that LL-37 promoted EMT, migration and invasion of HCC cells via MAPK/ERK signaling. Actually, the MAPK/ERK signaling pathway plays an important role in tumor invasion and metastasis [49]. ERK1/2 activation has also been linked to TGF-β-induced EMT and cell invasiveness [31]. Other signaling pathways which promote migration and invasion induced by LL-37 have been reported in other malignant tumors, such as the NF-κB pathway in melanoma cells [15] and the MAPK/ERK pathway in breast cancer and prostate cancer cells [13,17]. A study reported that LL-37 enhanced invasion, metastasis and tumorigenesis through FPR2 and P2X7 in pancreatic cancer stem cells [14]. Receptor tyrosine kinases are key factors lying upstream of the MAPK pathway. Our previous data revealed that the receptor tyrosine kinases HER2/EGFR were targets for LL-37 in HCC cells. Here, inhibiting the phosphorylation of HER2/EGFR by neratinib significantly inhibited LL-37-induced EMT, migration and invasion. Therefore, the HER2/EGFR-MAPK/ERK pathway participates in LL-37-induced EMT and migration of HCC cells. Actually, several pathways are related to the effect of LL-37 overexpression in HCC cells, including the PI3K/Akt, MAPK and JAK/STAT pathways [19]. However, whether these signalling pathways are also involved in the migration and invasion of HCC cells were not examined in our current study, further study is needed.

Although hCAP18/LL-37 was down-regulated in human HCC cells and HCC tumors [19], results from our si-LL-37 experiment confirmed that low LL-37 levels may be sufficient to promote the EMT, migration and invasion of HCC cells. Moreover, under special circumstances such as 1,25(OH)_2_D_3_ treatment for cancer, microbial infections and UVB ultraviolet light, the LL-37 level will be significantly increased [50–52]. Here we found that 1,25(OH)_2_D_3_ significantly induced VDR expression and nuclear transport, as well as hCAP18/LL-37 expression. The mesenchymal-like phenotype could revert to an epithelial-like phenotype in HCC cells caused by 1,25(OH)_2_D_3_ resulting from the increased E-cadherin level, which is consistent with previous evidence [25]. Our study further revealed that 1,25(OH)_2_D_3_ significantly decreased N-cadherin, Vimentin, MMP9 and MMP14, in conjunction with inhibition of migration and invasion of HCC cells. More interestingly, 1,25(OH)_2_D_3_ treatment together with knockdown of LL-37 further enhanced 1,25(OH)_2_D_3_-induced inhibition of migration and invasion by HCC cells, along with enhanced reversion of the EMT process in HCC cells. As mentioned above, LL-37 promoted EMT, migration, invasion and metastasis in HCC cells. Although the detailed mechanism by which 1,25(OH)_2_D_3_ inhibits HCC metastasis is unclear, here for the first time, we revealed that hCAP18/LL-37 may be an important factor which suppresses the therapeutic benefit of 1,25(OH)_2_D_3_ in HCC tumors by promoting metastasis, which further supports the hypothesis that hCAP18/LL-37 may be an important target which can improve the anticancer activity of 1,25(OH)_2_D_3_ in HCC therapy.

## Conclusions

In conclusion, for the first time to our knowledge, our study shows that LL-37 promotes EMT, migration and lung metastasis of HCC cells in vitro and in vivo. The HER2/EGFR-MAPK/ERK signaling pathway mediates LL-37-induced EMT, migration and invasion (Figure 7c). We believe that in addition to promoting tumor growth, LL-37 can also play an important role in HCC tumor progression by promoting HCC metastasis. Additionally, LL-37 may be an important factor interfering with 1,25(OH)_2_D_3_ inhibition of HCC cell metastasis. Therefore, 1,25(OH)_2_D_3_ treatment combined with silencing hCAP18/LL-37 expression may be a potential strategy to increase the anticancer activity of 1,25(OH)_2_D_3_ in treating HCC progression.

## Supplementary Material

Supplemental Material

## Data Availability

The authors confirm that the data supporting the findings of this study are available within the article.
