# Supplementary material for: Cathelicidin LL-37 promotes EMT, migration and metastasis of hepatocellular carcinoma cells in vitro and mouse model
Source: Cell Adh Migr. 2023 Jan 19;17(1):20–34. doi: 10.1080/19336918.2023.2168231 (PMC9858423; doi:10.1080/19336918.2023.2168231)
Supplement: Supplemental Material [file KCAM_A_2168231_SM3854.docx]

### Cathelicidin LL-37 promotes EMT, migration and metastasis of hepatocellular carcinoma cells in vitro and mouse model

Huidan Zhang^1^, Xueli Yuan^1^, Yaxin Yang^1^, Yangke Wanyan, Liping Tao^1^, Yuqing Chen^1*^.

^1^Jiangsu Province Key Laboratory for Molecular and Medical Biotechnology, Life Sciences College, Nanjing Normal University, Nanjing, China.

* Correspondence: Yuqing Chen

Jiangsu Province Key Laboratory for Molecular and Medical Biotechnology, Life Sciences college, Nanjing Normal University, 1# Wenyuan Rd, Nanjing 210000, Jiangsu Province, PR China.

Tel +13645197488

Fax +8602586227805.

Email address: [chenyuqing@njnu.edu.cn](mailto:chenyuqing@njnu.edu.cn)

**
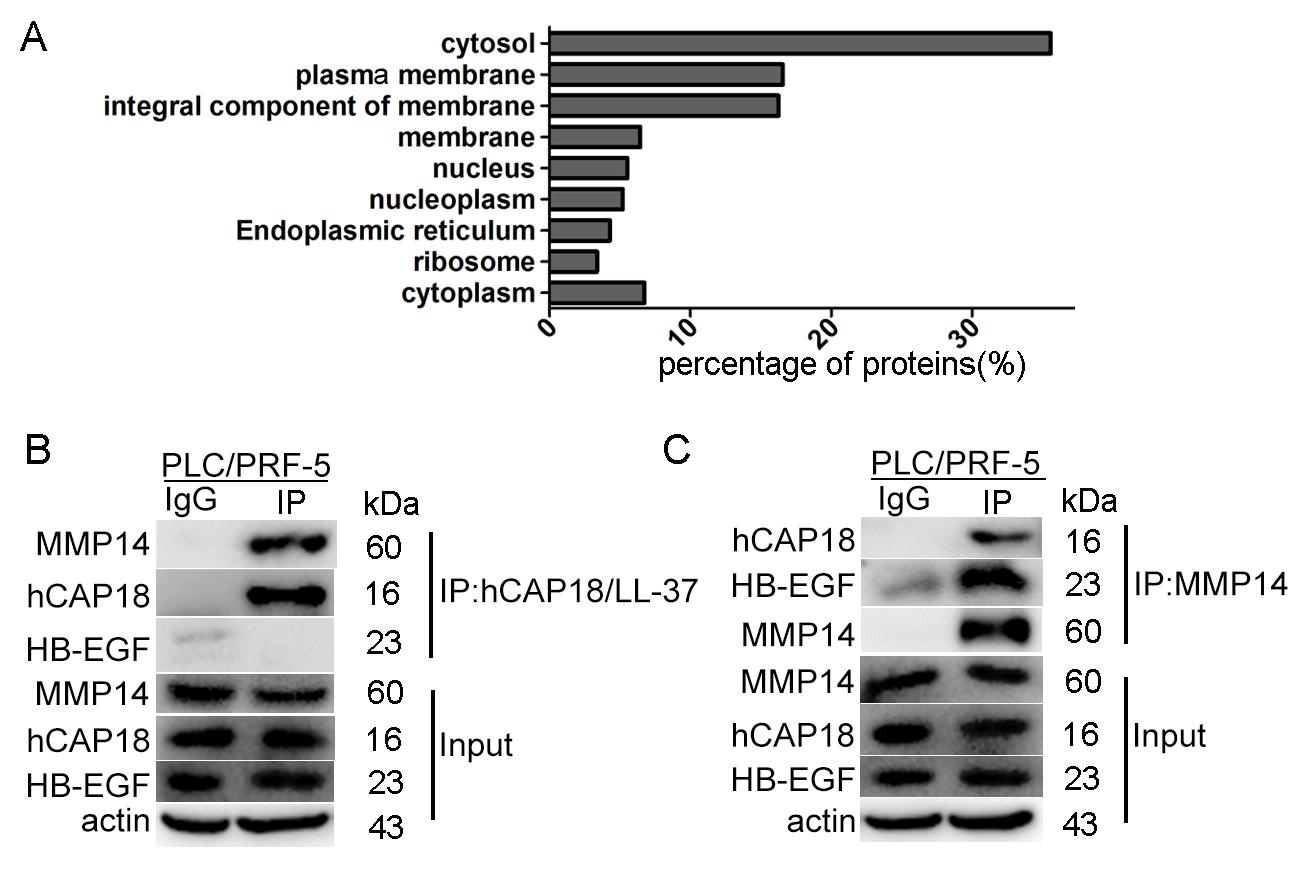
**

**Figure S1. Identification of the interaction between MMP14 and hCAP18.** A. PLC/PRF-5 cells treated with LL-37 were subjected to Co-IP using hCAP18/LL-37 antibody and Protein A/G agarose beads, then analyzed by mass spectrometry. The percentage of proteins shown to interact with hCAP18/LL-37 are indicated by GO analysis. PLC/PRF-5 cells were treated with LL-37 for 48 h before being harvested for preparation of whole-cell extracts, and the resulting extracts were used to conduct Co-IP assays using anti-hCAP18/LL-37 (B) or anti-MMP14 antibody (C). Western blot assays were then performed using anti-MMP14, anti-HB-EGF or anti-hCAP18/LL-37.
